# Isolation of *Chlamydia abortus* from a laboratory worker diagnosed with atypical pneumonia

**DOI:** 10.1186/s13620-016-0067-4

**Published:** 2016-07-20

**Authors:** Nieves Ortega, M. Rosa Caro, M. Carmen Gallego, Antonio Murcia-Belmonte, Daniel Álvarez, Laura del Río, Francisco Cuello, Antonio J. Buendía, Jesús Salinas

**Affiliations:** 1 Departamento de Sanidad Animal, Facultad de Veterinaria, Campus Regional de Excelencia internacional, Campus Mare Nostrum, Universidad de Murcia, Murcia, Spain; 2 Departamento de Anatomía y Anatomía Patológica Comparadas, Facultad de Veterinaria, Campus Regional de Excelencia internacional, Campus Mare Nostrum, Universidad de Murcia, Murcia, Spain

**Keywords:** *Chlamydia abortus*, Human, Atypical pneumonia, Zoonoses, OEA

## Abstract

**Background:**

Identifying the aetiological agent of atypical pneumonia in human can sometimes be a tedious process, especially in cases where *Mycoplasma pneumoniae*, *Legionella* species and *Chlamydia pneumoniae* are ruled out. In such cases, a correct anamnesis of the patient is basic to clarify which pathogens might have produced the infection. For this reason, health professionals including veterinarians and laboratory personnel working with zoonotic pathogens should keep their doctors informed.

**Case presentation:**

A human case of atypical pneumonia linked to *Chlamydia abortus* is reported. A 47-year-old male, a veterinarian researcher into chlamydiae, developed respiratory symptoms, breathing problems and high fever. Serological analyses ruled out the involvement of several respiratory pathogens, such as *M. pneumoniae*, *Legionella pneumophila*, *Rickettsia conorii* and *C. pneumoniae,* and *Chlamydia abortus* was identified as the possible aetiological agent of the infection. The isolation of *C. abortus* from the patient’s sputum and subsequent molecular analysis confirmed the presence of this microorganism.

**Conclusion:**

As far as we know, although *C. abortus* has not been previously described as capable of causing pneumonia in humans, this is the first reported case of atypical pneumonia in which *C. abortus* is thought to have played an aetiological role.

## Background

The term ‘atypical pneumonia’ is applied to pneumonia caused by *Mycoplasma pneumoniae, Legionella* species and *Chlamydia pneumoniae,* although some authors argue that other emerging atypical pneumonia pathogens should be included in the list [1].

The Family *Chlamydiaceae* includes 11 species grouped in a single genus, *Chamydia* [2]. These are Gram-negative, obligate intracellular bacteria with a unique bi-phasic developmental cycle, in which a small extracellular infectious elementary body (EB) and a metabolically active reticulate body (RB) play **s**pecial roles. Chlamydial infections cause clinically and epidemiologically important diseases both in humans and in animals [3]. Respiratory tract infections and atypical pneumonia in humans are commonly associated with *Chlamydia pneumoniae* infections [4]. *C. psittaci* is the causative agent of psittacosis or ornithosis in birds. This disease is a zoonosis transmitted through aerosols that especially affects workers who come into contact with domestic poultry or wildfowl. The clinical course of this infection in humans starts with influenza-like symptoms, progresses as atypical pneumonia and may lead to systemic infections with serious complications in internal organs [5].

*C. abortus* infection is also considered as a zoonotic pathogen with serious effects for infected pregnant women and unborn children [6]. This bacterium is associated with enzootic abortion in small ruminants (OEA), and can also infect other animal species such as pigs, cattle and wild ungulates [3, 5, 7, 8]. To our knowledge, this is the first time that *C. abortus* has been reported as the likely causative agent of atypical pneumonia in humans.

## Case Presentation

A 47-year-old male reported a seven day history of influenza-like symptoms, including general malaise, chills, dry cough, chest-pain, shortness of breath, and temperatures of up to 39.5 °C, which did not respond to paracetamol or ibuprofen. The patient worked as a veterinary researcher in a laboratory where experimental intranasal infections with *C. abortus* were developed in sheep. The patient carried out these infections using a suspension of *C. abortus* sprayed into both nostrils with a multi-dose spray pump, as described previously [9]. Ten days after the sheep infections, the patient showed the first symptoms. 

**Fig. 1:**
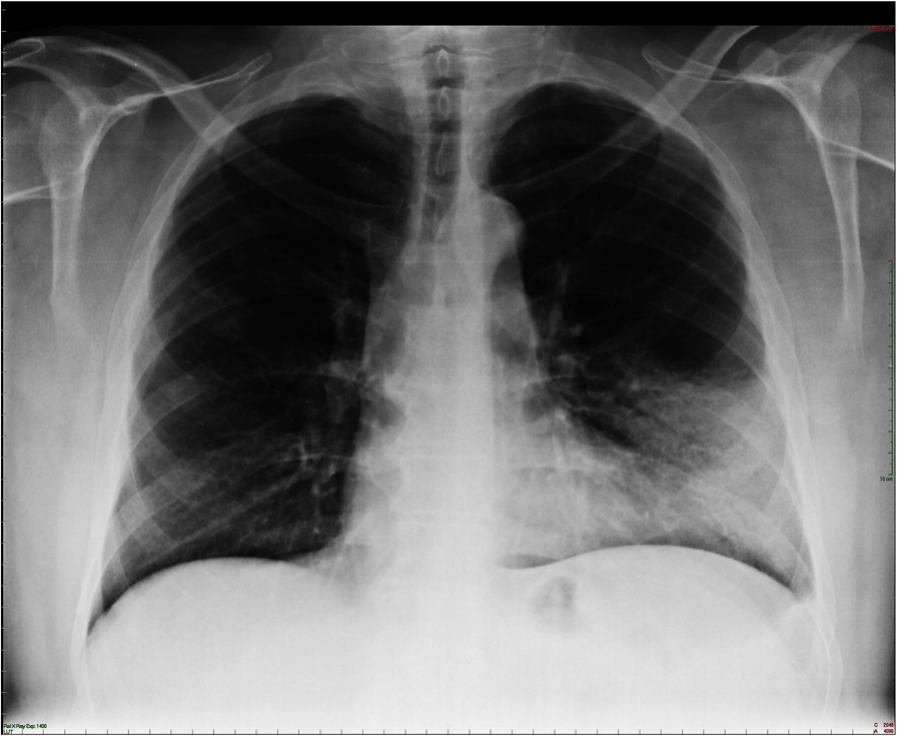
Chest x-ray of the patient with dense infiltration in the left lower lobe

The initial medical examination identified crackling sounds during lung auscultation. A chest x-ray showed areas of consolidation of the lower lobe of the left lung, indicating a pan-lobar pneumonia (Fig. 1). Blood and biochemical tests revealed a non-significant increase in mean corpuscular hemoglobin (MCH), as well as a slight increase in urea and creatinine levels: 50.3 and 1.3 mg/dl respectively. By contrast, the C-reactive protein level was very high, 8.9 mg/dl, normal values being <0.5 mg/dl. These high values suggested a bacterial infection rather than a viral infection, in which case the values would be lower [10].

Serological analyses with several ELISA tests (all of them from Vircell Microbiologist, Granada, Spain) using specific IgG and IgM monoclonal antibodies (MoAb) were negative against *Mycoplasma pneumoniae*, *Legionella pneumophila* (serogroup 1-7), *Rickettsia conorii* and *Chlamydia pneumoniae*.

Due to the suspicion of *C. abortus* infection, serum and sputum samples from the patient were sent to a laboratory at the Animal Health Department of the Murcia University (Spain) for further analysis.

A *C. abortus*-specific serological test, performed using an ELISA kit (ID Screen *Chlamydophila abortus* indirect multi-species, IDvet), revealed high IgM and IgG optical densities (OD) in the serum samples (Table 1). The serum samples were tested with a peroxidase-conjugated anti-human IgG or IgM (Sigma), and the OD were determined (DigiScan with DigiWin Software, ASYS Hitech). In addition, two serum samples from asymptomatic work colleagues were analyzed with the same ELISA kit. As might be expected, both colleagues showed antibody production against *C. abortus*, but with a lower OD than the patient, especially for IgM production (Table 1). It was therefore assumed that antibody production on the part of the patient was due to a clinical episode and not to a progressive sensitization to *C. abortus*.

*C. abortus* was isolated from the sputum using a McCoy cell monolayer, as described previously [11]. Positive cell cultures were identified by an immunofluorescence assay using the *C. abortus*-specific anti-MOMP FA2H10 MoAb [12]. The MoAb was produced using BALB/c mice and purified by immunoafinity, and was chosen because it is specific for an oligomer of the outer membrane protein of *C. abortus* (named as *C. psittaci*, serotype 1).

*C. abortus* DNA was detected in the sputum sample and also in the cell culture isolates by PCR analysis. Total DNA was extracted with a commercial kit (DNeasy tissue kit, Qiagen) and was used as a template for a *C. abortus*-specific PCR, using primers pmp-F (5′-CTC ACC ATT GTC TCA GGT GGA-3′) and pmp-R821 (5′-ACC GTA ATG GGT AGG AGG GGT-3′) for the target gen *pmp 90/91* [13], which allow the sensitive amplification of 821-bp length DNA fragments. In order to confirm the specific presence of *C. abortus* in the sputum sample as well as in the cell isolates, the amplicons were sequenced (ABI Prism 3130). The PCR products showed 100 % identity to the *C. abortus* AB7 strain sequence obtained using MEGA5 software and BLAST® program. This *C. abortus* strain was the same strain as that used in the experimental infections in ewes carried out by the patient [9].

Once diagnosed, the patient was treated with antibiotics (levofloxacin, initial intravenous dose of 500 and 500 mg orally every 24 h for 10 days; and clarithromycin 500 mg every 12 h for 10 days), mucolytic carbocysteine lysine (2.7 g orally every 24 h), glucocorticoids (beclomethasone dipropionate inhaler 100 μg every 12 h) and non-steroidal anti-inflammatory drugs (paracetamol 1 g orally in case of fever or pain). This treatment resulted in successful recovery of the patient in 2 weeks without sequels.

**Table 1:** IgG and IgM levels against *C. abortus* in serum samples from the patient and two asymptomatic work colleagues (Researcher 1 and Researcher 2) expressed as optical densities (OD) by ELISA

	IgG (OD)	IgM (OD)
Patient	1.746	0.627
Researcher 1	1.235	0.137
Researcher 2	0.866	0.112

## Discussion

Atypical pneumonia caused by *Chlamydia* species, mainly *C. pneumoniae* and *C. psittaci* infections*,* has frequently been commonly reported in human patients [1]. In Spain, published data revealed the presence of *C. pneumoniae* in 14.3 % of pneumonia inpatients and in 25 % of outpatient cases, while *C. psittaci* was detected in 0.7 % of hospitalized pneumonia cases [14]. However, there is no previous published evidence that *C. abortus* can infect the human respiratory tract. Previous published research involving experimental infections with *C. abortus* has identified the ability of this pathogen to cause pulmonary disease in animal species such as sheep [9, 15], calves [16] and mice [17]. The detection of *C. abortus* in a broncho-alvolar lavage (BAL) would have been decisive to firmly confirm the etiological agent of the process. However, although this procedure was not performed, the findings observed in this report may suggest the role of *C. abortus* as the aetiological agent in this case of human pneumonia.

*C. abortus* is typically associated with abortive disorders in small ruminants and other animal species [3, 7, 8]. It is also known as a zoonotic agent, affecting pregnant women who have been in contact with infected animals [6, 18]. Therefore, farmers, veterinarians and other professionals who may come into contact with chlamydia should be alert when managing vaginal discharges, placentas from aborting females or aborted fetuses because of the risk of oronasal entry of *C. abortus*.

Marrie et al. [1] reviewed the role of atypical pathogens in community-acquired pneumonia and categorized new emerging atypical pneumonia agents. The present report suggests that *C. abortus* should be included within this group and should be taken into account for differential diagnoses in respiratory patients with a history of contact with aborted sheep or goats or their afterbirth.

Human chlamydial pneumonia is not usually a life-threatening condition and patients, following correct treatment, usually recover. Nevertheless, they should be closely monitored because chlamydiae can cause serious complications in internal organs after systemic infections [5, 18]. Treatments are similar for all chlamydial infections. So, in the present case, broad-spectrum antibiotic, mucolytic and glucocorticoid treatment led to a successful recovery with no pulmonary sequels.

## Conclusions

In summary, the first suspected case of *C. abortus* zoonotic respiratory infection is reported, whereby, instead of being associated with reproductive problems, it is described as the probable causative agent of human atypical pneumonia. So, veterinarians, veterinary laboratory personnel, and public health officials should be aware of possible pulmonary infection due to *C. abortus* aerosol inhalation.

## Abbreviations

BAL, broncho-alvolar lavage; DNA, deoxyribonucleic acid; EB, elementary body; ELISA, enzyme like immunoabsorbent assay; IgG, immunoglobulin G; IgM, immunoglobulin M; MCH, mean corpuscular hemoglobin; MoAb, monoclonal antibodies; MOMP, major outer membrane protein; OD, optical densities; OEA, ovine enzootic abortion; PCR, polimerase chain reaction; RB, reticulate body
